# Impact of Sinbaglustat on Neurons of the Medial Nucleus of the Trapezoid Body in a Murine Model of Human G_M1_-Gangliosidosis

**DOI:** 10.3390/jcm15062249

**Published:** 2026-03-16

**Authors:** Lorna Jubran, Rouven Wannemacher, Wolfgang Baumgärtner, Felix Felmy, Michel Alexander Steiner, Eva Leitzen, Nikolaos Kladisios

**Affiliations:** 1 Department of Pathology, University of Veterinary Medicine, 30559 Hannover, Germany; lorna.jubran2@gmail.com (L.J.); rouven.wannemacher@tiho-hannover.de (R.W.); eva.leitzen@tiho-hannover.de (E.L.); 2 Center for Systems Neuroscience, Hannover Graduate School for Neurosciences, Infection Medicine, and Veterinary Sciences (HGNI), 30559 Hannover, Germany; 3 Institute of Zoology, University of Veterinary Medicine, 30559 Hannover, Germany; felix.felmy@tiho-hannover.de (F.F.); nikolaos.kladisios@tiho-hannover.de (N.K.); 4 Idorsia Pharmaceuticals Ltd., 4123 Allschwil, Switzerland; michel.steiner@pharvaris.com

**Keywords:** G_M1_, MNTB, electrophysiology, electroporation, substrate reduction therapy, sinbaglustat

## Abstract

**Background**: G_M1_-gangliosidosis (G_M1_) is a lysosomal storage disorder caused by mutations in the *Glb1* gene, resulting in reduced β-galactosidase activity and accumulation of G_M1_ gangliosides in neuronal lysosomes. Effective therapeutic strategies for this disease remain limited. Substrate reduction therapy using small molecules targeting glucosylceramide synthase (GCS) and non-lysosomal glucosylceramidase (GBA2), such as sinbaglustat, represents a promising approach. **Methods**: Structural and electrophysiological properties of principal neurons of the medial nucleus of the trapezoid body (MNTB) were investigated in 7-month-old *Glb1*^−/−^ mice. Animals received long-term treatment with either low (LD; 10 mg/kg) or high (HD; 300 mg/kg) doses of sinbaglustat and were compared with untreated *Glb1*^−/−^ (KO) and untreated wild-type (WT) mice. **Results**: Sinbaglustat treatment reduced lysosomal storage material in MNTB neurons. Basal membrane properties were largely unchanged across groups. However, action potential halfwidth was significantly increased in untreated KO and LD mice compared to untreated WT animals but was normalized in HD mice. After-hyperpolarization duration was prolonged in *Glb1*^−/−^ mice relative to WT. Temporal precision during high-frequency stimulation was reduced in untreated KO mice and improved following sinbaglustat treatment. **Conclusions**: These findings indicate that G_M1_-gangliosidosis is associated with functional alterations in MNTB neurons and suggest that long-term sinbaglustat treatment can partially restore neuronal electrophysiological properties, supporting its therapeutic potential in G_M1_.

## 1. Introduction

G_M1_-gangliosidosis is a lysosomal storage disease (LSD) caused by a mutation in the *Glb1* gene, resulting in β-galactosidase enzyme (β-gal) deficiency [1] and progressive accumulation of the ganglioside G_M1_ and its derivate GA1 in lysosomes [2,3]. The accumulation is particularly pronounced in neurons, where gangliosides are incorporated into the cell membrane up to ten times more frequently than in other mammalian cells [4,5]. This accumulation negatively affects various biochemical pathways [6] and may lead to neurodegeneration and premature death [7]. The reported symptoms in human patients vary depending on the clinical course and exhibit a high phenotypic variability including impairment of the visual and auditory systems [8]. In addition to humans, G_M1_-gangliosidosis has been described in various mammals, including cats [9,10], dogs [11,12] and ruminants [13], among others. Moreover, various mouse models have been established [8,14,15,16] to develop potential therapeutic strategies.

Several experimental methods for the treatment of G_M1_-gangliosidosis have been developed, including therapy with GBA2 and GCS inhibitors [17,18], enzyme replacement [19] and gene therapy [20]. The principle behind inhibiting GCS, the main enzyme involved in the initial step of the biosynthesis of gangliosides, is substrate reduction therapy (SRT), which aims to reduce the accumulation of the storage material [8,21]. To effectively manage the symptoms of G_M1_-gangliosidosis with SRT, small molecules must be able to permeate the blood–brain barrier, where G_M1_ accumulation is most severe [8]. This is achieved with the drugs that have been deployed so far, including miglustat [17], venglustat [22], lucerastat [23], and sinbaglustat [24]. Interestingly, miglustat, lucerastat and sinbaglustat, besides inhibiting GCS, are also potent inhibitors of GBA2, an enzyme that catabolizes a sphingolipid pool outside of the lysosome [25] and might thereby have a positive indirect influence on the function of lysosomes in the context of lysosomal storage disorders (LSDs) [26].

Sinbaglustat is a brain-penetrating, orally bioavailable iminosugar that has a dose-dependent dual-inhibitory effect on GBA2 and GCS [27,28]. Since the potency of sinbaglustat for inhibiting GBA2 is greater than for inhibiting GCS [27], higher doses that additionally inhibit GCS, along with GBA2, are expected to prevent excessive G_M1_ accumulation in lysosomes [28]. However, data regarding the efficacy of sinbaglustat on the single-cell level are missing.

A suitable system to study features of auditory impairment and the efficacy of treatment on biophysical parameters of neurons is the medial nucleus of the trapezoid body (MNTB). The rather homogenous population of neurons of the MNTB sign-inverts excitatory inputs of the calyx of Held to glycinergic inhibition [29], targeting other ipsilateral ascending auditory structures [30]. This large calyceal synapse can fire action potentials (APs) with high fidelity up to 1 kHz [31,32]. Furthermore, the expression of low- and high-voltage-activated potassium currents shapes the firing waveform [33,34,35], resulting in single onset responses with narrow APs. A hallmark of this one-to-one input–output function is its high temporal precision that can be observed in vivo [36,37].

The present study investigated the effect of sinbaglustat on the lysosomal storage of G_M1_ and functional parameters of MNTB neurons of *Glb1*^−/−^ transgenic mice using ex vivo transverse brain slices. Cytoplasmic vacuolization in neurons of the MNTB was assessed by electroporation, while the biophysical changes in an advanced stage of G_M1_-gangliosidosis were analyzed with whole-cell, patch-clamp electrophysiology.

## 2. Materials and Methods

### 2.1. Animals

Wild-type (WT) and *Glb1^−/−^* transgenic mice on a C57BL/6 genetic background were generated as part of a previous in-house study [16]. The corresponding genotype of the animals was determined by PCR tests as described previously [16]. Six age-matched male and female *Glb1^−/−^* mice were randomly assigned to three treatment groups (see below; *n* = 18). In addition, six wild-type (WT) mice were used as a control group (total number of animals: *n* = 24). All animals were included in the final analysis. Animals were kept in isolated ventilated cages with ad libitum access to food and water [38].

*Glb1^−/−^* mice were either untreated (KO; standard diet: Kliba Extrudate Ref 3336) or fed with a diet containing 10 mg/kg (low dose; LD) or 300 mg/kg (high dose; HD) of sinbaglustat, respectively, starting at 4–5 weeks of age. Daily sinbaglustat intake was monitored by correlating the average amount of food available in the cage with the body weight of the animals. At 6 months of age, the effective average daily intake was 8.51 mg/kg for LD-treated and 235 mg/kg for HD-treated *Glb1^−/−^* mice [38]. WT animals received the same feeding as untreated *Glb1^−/−^* mice (standard diet: Kliba Extrudate Ref 3336). A second group of WT animals treated with HD was included and analyzed in parallel. Since no significant differences were found compared to untreated WT animals, these data were not included in the present study. Moreover, potential confounders (e.g., treatment order, measurement order, or cage location) were not applicable, as all analyses were conducted post mortem.

This study was semi-blind with regard to the treatment of *Glb1*^−/−^ mice (the respective concentrations for the treatment groups were blinded, so that the experimenters did not know whether the animals were receiving one of the two doses of sinbaglustat or the standard feed).

All animal experiments were performed in accordance with the German Animal Welfare Law and were approved by the local authorities (Niedersächsisches Landesamt für Verbraucherschutz und Lebensmittelsicherheit (LAVES), Oldenburg, Germany, permission number 33.8-42502-21/3632; 20 May 2021) and complied with ARRIVE guidelines.

### 2.2. Slice Preparation and Electrophysiology

Mice were sacrificed at 7 months of age and prepared for electrophysiological experiments and single-cell electroporation, as described in previous electrophysiological studies on this mouse strain [16]. Brains were quickly transferred to an ice-cold slice solution containing the following (in mM): 30 NaHCO_3_, 1.2 NaH_2_PO_4_, 2.5 KCl, 25 glucose, 20 HEPES, 93 NMDG, 5 ascorbic acid, 3 myo-inositol, 3 Na-pyruvate, 10 MgCl_2_, 2 CaCl_2_ and 93 HCl. To ensure oxygenation, the slice solution was continuously bubbled with 95% oxygen and 5% carbon dioxide, maintaining a pH of 7.4. Transversal brain slices of 200 µm thickness at the level of the MNTB were prepared with a VT1200S vibratome (Leica VT1200, Leica Microsystems GmbH, Wetzlar, Germany). Brain slices were subsequently incubated in the slice solution for 7 min at 37 °C before being transferred to an oxygenated recording solution at room temperature containing the following (in mM): 125 NaCl, 25 NaHCO_3_, 2.5 KCl, 1.25 NaH_2_PO_4_, 1 MgCl_2_, 1.2 CaCl_2_, 25 glucose, 0.4 ascorbic acid, 3 myo-inositol and 2 Na-pyruvate. Whole-cell recordings were obtained from living neurons of the MNTB, which were visualized with either a CCD-camera (TILL-Imago VGA, Retiga 2000DC; TILL Photonics GmbH, Planegg, Germany) controlled by TILLvisIONimaging system (TILL Photonics GmbH, Planegg, Germany), or with a pco.edge 3.1 camera (Excelitas Technologies, Waltham, MA, USA). Recordings were performed using an EPC10/2 amplifier controlled by PatchMaster (HEKA Elektronik GmbH, Lambrecht/Pfalz, Germany). All voltage potentials were corrected offline for liquid junction potential (LJP) of 16 mV, which was estimated according to custom-written scripts. To minimize artifacts arising from pipette capacitance, capacitance compensation was applied prior to both voltage- and current-clamp recordings. Glass pipettes with a resistance between 3 and 5 MΩ were filled with an internal solution consisting of the following (in mM): 145 K-gluconate, 4.5 KCl, 15 HEPES, 2 Mg-ATP, 2 K_2_-ATP, 0.3 Na_2_-GTP, 7 Na_2_-phosphocreatine, 0.5 K-EGTA and 0.05 Alexa-594. Data were collected with a sample rate of either 50 or 100 kHz and filtered at 3 kHz. Access resistance was estimated under voltage clamp before switching to current clamp configuration with the bridge balance set to 100%.

#### 2.2.1. Membrane Properties

To estimate the somatic capacitance (C), 100 repetitions of a −5 mV step potential were applied and averaged, and a biexponential curve was fitted at onset. The integrated area between the onset of the stimulus and five times the weighted decay time constant, which corresponds to the capacitive charge, was used to calculate C. To calculate the input resistance (R_in_) close to resting potential, 100 repetitions of −5 pA hyperpolarizing current were averaged and Ohm’s law was applied at steady state. The membrane time constant (t_mem_) was estimated by fitting an exponential function at stimulus onset of the averaged trace. The resting potential (E_rest_) was estimated from the same trace before current injection.

#### 2.2.2. Suprathreshold Response Analysis

Action potentials were elicited by injecting a ramp current with 0.2 ms rise and 0.8 ms descending duration increased in 50 pA intervals. From the first suprathreshold event, a phase plane plot of dV/dt against V was plotted to assess the voltage threshold (V_thres_), which was defined by a rapid increase in dV/dt. Action potential amplitude was defined as the distance from the resting potential to the peak of the action potential, and halfwidth as the duration between the rise and fall level equal to half the distance between the voltage threshold and the action potential peak. To estimate the after-hyperpolarization halftime duration, a mono-exponential curve was fitted at the minimum voltage level after the action potential.

To assess the temporal precision of MNTB neurons, 100 repetitions of a 500 ms-long square current were injected at rheobase, leading to a mix of sub- and suprathreshold responses. Latency was estimated as the mean duration between stimulus onset and maximum action potential deflections, and jitter as the standard deviation of latency. Finally, ramp currents of 30 pulse trains at a frequency of 500 Hz were injected and adjusted, until all steps could reliably generate output. The median latency and jitter (as standard deviation of the latency) of each step was calculated for each animal group.

#### 2.2.3. Single-Cell Electroporation

For single-cell electroporation, a patch pipette was loaded with 10 mM Alexa Fluor™ 568 sodium salt hydrazide and was pushed on the surface of the visually identified MNTB neuron. The dye was loaded into the neuron by a 10–15 ms long, 10–15 V voltage pulse. To confirm successful single-cell labeling, the fluorescence was monitored online using a monochromator system (Polychrome IV; Till Photonics GmbH, Planegg, Germany). Subsequently, slices containing labeled neurons were fixed in 4% paraformaldehyde overnight. Following fixation, slices were washed three times for 5 min with PBS, mounted in VECTASHIELD® mounting medium (H-100, Vector Laboratories Inc., Newark, CA, USA) and placed under a coverslip sealed with nail polish. Afterwards, confocal image stacks of labeled cells were performed using a SP5 System (Leica Microsystems GmbH, Wetzlar, Germany) with a 63×/1.4NA oil immersion objective. The x- and y-steps of the confocal microscope were 320 nm, and the z-step size 290 nm.

To analyze images, sequences were imported into Fiji 2.14.0, and the color scheme was standardized by converting images to 8-bit grayscale. A region of interest (ROI) with a size of 100 × 100 pixels was centered on a selected cell and cropped for further analysis. The cropped image was used to generate a maximum projection of three consecutive planes, starting from the level where the nucleus was clearly discernible. The soma of the selected cell was selected, the surrounding of the cell was erased, and a fluorescence intensity profile was generated. In the intensity profile, the second highest peak, corresponding to the cytoplasmic region, was defined as the maximum threshold for each individual cell. Afterwards, binary images were generated, and the pixel numbers were extracted. The vacuolization rate was calculated as the ratio of black to white pixels of threshold images.

### 2.3. Data Analysis and Statistics

Data analysis was performed using custom-written functions in Igor Pro 9 (Wavemetrics, Lake Oswego, OR, USA) and Microsoft^®^ Excel^®^. The dataset of the electroporated neurons was taken from 6 wild-type (WT), 20 non-treated *Glb1^−/−^*, 20 *Glb1^−/−^* LD treated, and 20 *Glb1^−/−^* HD treated MNTB neurons, from 4, 5, 3 and 5 animals, respectively. The dataset for the electrophysiological analysis included recordings from 27 WT, 19 non-treated *Glb1^−/−^*, 24 *Glb1^−/−^* LD and 24 *Glb1^−/−^* HD neurons, from a total of six animals from each group. Statistical analysis was conducted using SPSS^®^ for Windows^®^ version 29.0.1.0 (IBM^®^ SPSS^®^ Statistics, SPSS Inc.; Chicago, IL, USA). Normal distribution was tested with Shapiro–Wilk tests. Significant differences between treatment groups from the electrophysiological analysis were probed using Kruskal–Wallis tests and Dunn–Bonferroni post hoc tests. Statistical significance was set at 0.05.

## 3. Results

### 3.1. Sinbaglustat Treatment Reduces G_M1_ Accumulation in Principal MNTB Neurons of Treated Glb1^−/−^ Mice

**Figure 1:**
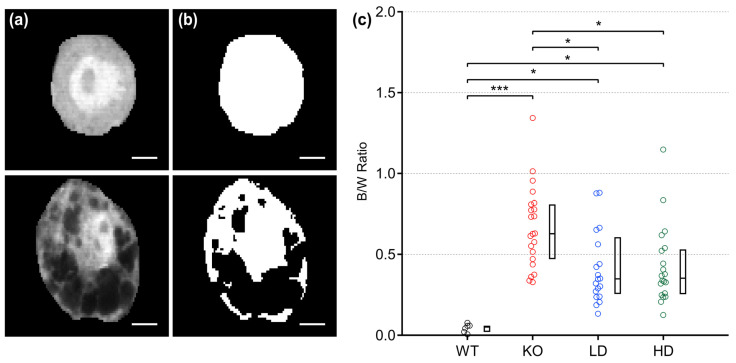
Sinbaglustat reduces vacuolization in principal MNTB neurons of *Glb1^−^^/−^* mice. (**a**) Intensity profiles of an exemplary electroporated untreated wild-type (WT; top) and untreated *Glb1^−/−^* (KO; bottom) MNTB neuron with different patterns of cytoplasmic dye distribution. The WT neuron displays a homogeneous, the *Glb1^−^^/−^* neuron an asymmetric staining pattern due to dye exclusion, indicative of extensive vacuolization. Scale bars: 5 µm. (**b**) After adjusting for intensity thresholds from individual cells, binary images of the corresponding neurons (top: WT; bottom: KO) were generated for quantification. Black areas correspond to vacuoles, where dye could not infiltrate. Scale bars: 5 µm. (**c**) The degree of vacuolization was assessed by calculating the ratio of black to white areas (B/W ratio) from individual cells. The graph shows color-coded individual data depending on the experimental group (WT; black, KO; red, low dose (LD); blue, high dose (HD); green) as well as the corresponding boxplots with median, first and third quartile. Statistical significance (Dunn–Bonferroni procedure following Kruskal–Wallis test) was accepted at *p* ≤ 0.05. n; WT = 6, KO = 22, LD = 20, HD = 20. *; *p* < 0.05, ***; *p* < 0.001.

*Glb1^−/−^* transgenic mice accumulate G_M1_ gangliosides in neuronal lysosomes, leading to enlargement of these lysosomes, which is visualized by dye exclusion [16]. To quantify the vacuolization at an advanced stage of the disease and to evaluate a potential therapeutic intervention with sinbaglustat, principal MNTB neurons were electroporated and the dye distribution was calculated. MNTB neurons of untreated WT animals demonstrated a homogeneously stained cytoplasm (Figure 1a, top). The lack of vacuolization was confirmed after image processing (Figure 1b, top). In MNTB neurons of untreated KO mice, the dye was not distributed evenly, and after image processing the vacuolization was estimated to cover only half of the somatic volume (Figure 1a,b, bottom). Threshold analysis (Figure 1c) revealed that untreated WT MNTB neurons displayed an even distribution of injected dye throughout the cytoplasm, reflected in a low black-to-white median ratio (0.05). Conversely, the neurons of untreated *Glb1^−/−^* (KO) mice showed marked cytoplasmic vacuolization with a median black-to-white ratio of 0.63 with a high range (0.33–1.34), confirming that, on average, more than half of the cytoplasmic volume was not filled with dye. A comparison of these values with the data from LD and HD mice showed that their average ratio was comparatively lower, with median values of 0.347 (range; 0.13–0.88) and 0.353 (range; 0.13–1.15), respectively. Regardless of the respective treatment group, KO mice showed more pronounced vacuolization of their neuronal cytoplasm than animals in the untreated WT group (Dunn’s post hoc test, WT vs. KO, *p* < 0.001; WT vs. LD, *p* = 0.012; WT vs. HD, *p* = 0.017). However, animals in the LD and HD groups showed significantly reduced vacuolization compared to KO animals (Dunn’s post hoc test, KO vs. LD, *p* = 0.040; KO vs. HD, *p* = 0.026). In contrast, there were no differences in the black/white ratio between LD and HD mice (Dunn’s post hoc test, KO vs. LD, *p* = 1.0. Therefore, a positive effect of sinbaglustat in reducing vacuolization in murine MNTB neurons was demonstrated.

### 3.2. Vacuolization Does Not Affect the Basal Membrane Properties of Murine Glb1^−/−^ MNTB Neurons

**Figure 2:**
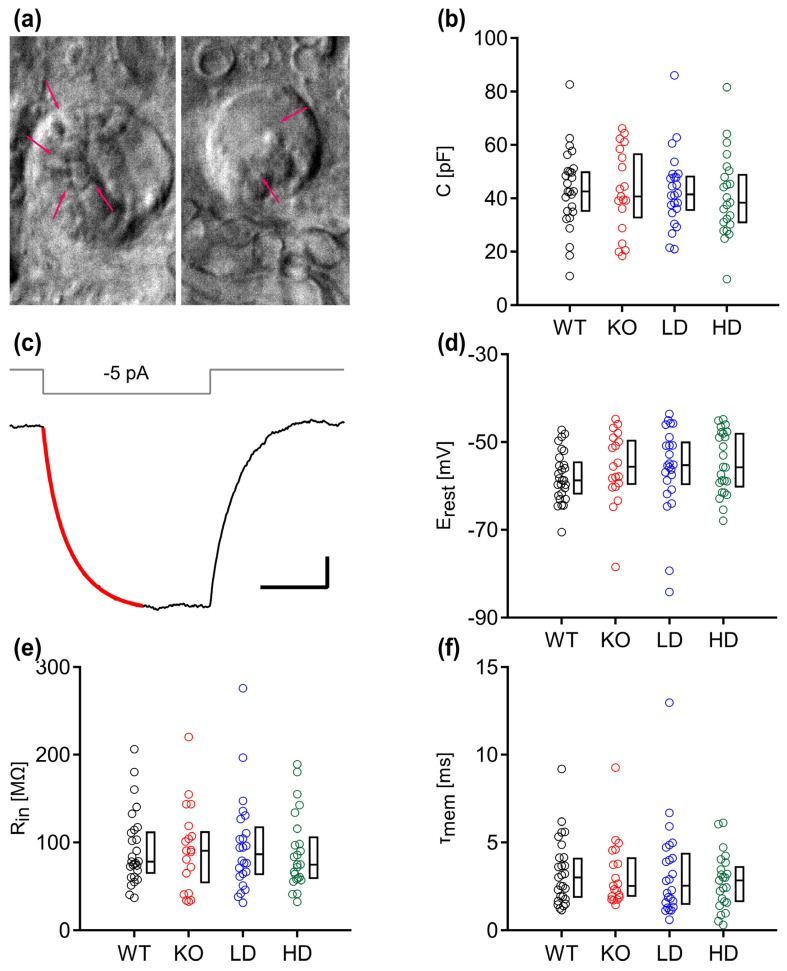
Basal membrane properties of principal MNTB neurons remain similar after sinbaglustat treatment. (**a**) The surface of a *Glb1^−/−^* MNTB neuron (left) has higher sphingolipid accumulation (arrows) than a low-dose treated neuron (right), indicated by the rougher morphology of the cell membrane of transgenic murine neurons. (**b**) The estimated somatic capacitance (C) of MNTB neurons was similar between animal groups. (**c**) Small subthreshold current injections (−5 pA) that induced hyperpolarization were used to extract the input resistance (R_in_). An exponential curve (red) was fitted at onset to estimate the membrane time constant (τ_mem_). Scale bars: 20 ms, 0.2 mV. (**d**–**f**) Resting potential (E_rest_), R_in_ and τ_mem_ of MNTB neurons did not differ between animal groups. The graphs show color-coded individual data depending on the experimental group (WT; black, KO; red, low dose (LD); blue, high dose (HD); green) as well as the corresponding boxplots with median, first and third quartile. Statistical significance (Dunn–Bonferroni procedure) was accepted at *p* ≤ 0.05. n; WT = 27, KO = 19, LD = 24, HD = 24.

To test whether the vacuolar accumulations and sinbaglustat treatment impact the biophysical properties of MNTB neurons, we performed patch-clamp electrophysiology on the MNTB of WT and *Glb1^−/−^* neurons. In agreement with the electroporation data, the cytoplasm of the neurons of KO mice appeared more irregular with cloudy aggregations, indicating intracytoplasmic accumulation of distended lysosomes (Figure 2a). The cell capacitance of individual neurons was calculated from −5 mV step-evoked charging transients and were similar between the groups (Figure 2b; Kruskal–Wallis, *p* = 0.155). The resting potential (E_rest_), input resistance (R_in_) and membrane time constant (τ_mem_) values were estimated by injecting current steps of −5 pA (Figure 2c–f). Likewise, these parameters did not differ between WT and KO or between KO and treated *Glb1^−/−^* mice (Kruskal–Wallis, E_rest_; *p* = 0.301, R_in_; *p* = 0.941, τ_mem_; *p* = 0.807).

### 3.3. Sinbaglustat Preserves the Waveform of Action Potentials in Treated Glb1^−/−^ Mice

**Figure 3:**
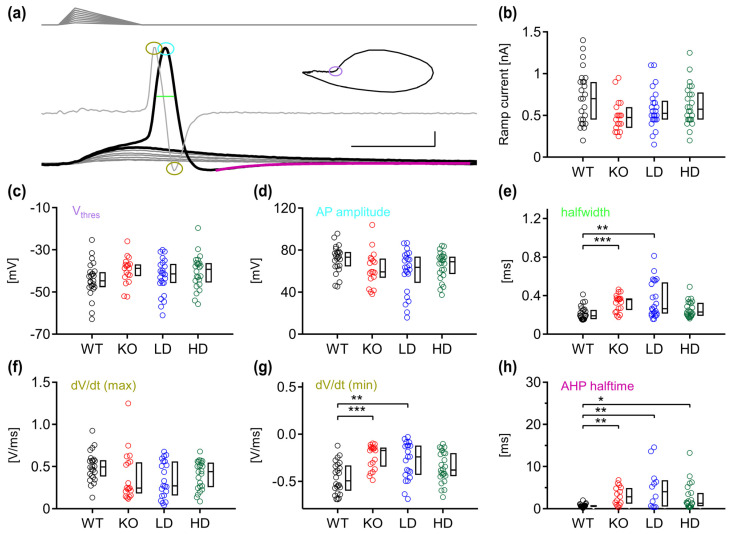
Action potential properties of sinbaglustat-treated and non-treated *Glb1^−/−^* mice have distinct differences. (**a**) Ramp stimuli (top) of increasing current were injected, and the voltage response was recorded (bottom). Subthreshold events are marked as gray traces, and the last sub- and first suprathreshold responses as black traces. The derivative of the first action potential was extracted (superimposed gray trace) and plotted against voltage (inset; phase plane plot), to calculate the voltage threshold (purple circle). The maximum AP amplitude (cyan circle) and halfwidth (light-green trace) were extracted from the first AP, and the maximum and minimum dV/dt values from the derivative (amber circles). The after-hyperpolarization halftime duration was calculated by fitting an exponential value at minimum deflection (magenta trace). Scale bars: 1 ms, 10 mV. (**b**) Ramp current injections that evoked the first suprathreshold event were similar between animal groups. (**c**–**e**) Voltage threshold and AP amplitude values were similar between groups. The AP halfwidth was significantly higher in the KO and LD group, compared to WT animals. (**f**,**g**) The maximum derivative values remain similar, but the minimum values differ between the WT group and the KO and LD animals. (**h**) After-hyperpolarization decay time values were lower in the WT group. The graphs show color-coded individual data depending on the experimental group (WT; black, KO; red, low dose (LD); blue, high dose (HD); green) as well as the corresponding boxplots with median, first and third quartile. Statistical significance (Dunn–Bonferroni procedure) was accepted at *p* ≤ 0.05. n; WT = 27, KO = 19, LD = 24, HD = 24. *; *p* < 0.05, **; *p* < 0.01, ***; *p* < 0.001.

Next, we investigated the action potential (AP) properties of MNTB neurons in WT and *Glb1^−/−^* mice before and after treatment by injecting ramp currents that generated a mix of sub- and suprathreshold events (Figure 3a). The ramp current needed to generate an AP did not significantly differ between the groups (Kruskal–Wallis, *p* = 0.082), although it was reduced in the untreated KO mice (KO; 475 pA, LD; 525 pA, HD; 575 pA) compared with the WT group (700 pA) (Figure 3b). Similarly, no significant difference was detected for the voltage threshold (V_thres_) (Kruskal–Wallis, *p* = 0.195) and maximum AP amplitude (Kruskal–Wallis, *p* = 0.108), even though the KO mice had the lowest median values (V_thres_ and AP amplitude, KO; −38.78 and 59.37 mV) and the treated *Glb1^−/−^* mice intermediate values (LD; −41.41 and 63.67 mV, HD; −39.27 and 69.01 mV) compared to the WT group (−44.72 and 73.39 mV) (Figure 3c,d).

We detected a significant difference in the AP halfwidth between the groups (Kruskal–Wallis, *p* < 0.001). The untreated KO mice had the largest median AP duration (0.358 ms), the treated groups intermediate (LD; 0.264 ms, HD; 0.229 ms), and the WT mice the lowest duration (0.194 ms) (Figure 3e). These halfwidth values differed significantly between WT and KO (Dunn’s post hoc test, *p* = 0.001) and WT and LD (Dunn’s post hoc test, *p* = 0.004), but not between WT and HD (Dunn’s post hoc test, *p* = 0.467). However, statistical significance was not reached between KO, LD and HD (Dunn’s post hoc test, KO vs. LD *p* = 1.0; KO vs. HD *p* = 0.179).

To further analyze the halfwidth broadening, the first derivative of the AP was extracted. The maximal acceleration of the AP (dV/dt max), which corresponded to the depolarization phase, did not show a significant difference between the animal groups (Kruskal–Wallis, *p* = 0.071; Figure 3f). However, the minimum dV/dt values differed significantly (Kruskal–Wallis, *p* = 0.001) (Figure 3g). The KO group had the least negative median value (−0.173 V/ms), followed by the LD (−0.24 V/ms), HD (−0.38 V/ms) and the WT group (−0.493 V/ms). As with the results of the AP halfwidth, we detected a difference in significance between WT and KO, and WT and LD but not between WT and HD (Dunn’s post hoc test, WT vs. KO, *p* = 0.001; WT vs. LD, *p* = 0.009; WT vs. HD, *p* = 0.355), as well as between KO, LD and HD (Dunn’s post hoc test, KO vs. LD, *p* = 1; KO vs. HD, *p* = 0.238; LD vs. HD, *p* = 1).

Finally, we analyzed the after-hyperpolarization kinetics by calculating the AHP decay time. Compared with WT mice, the KO, LD and HD groups had slower kinetics (Kruskal–Wallis: *p* = 0.001; Dunn’s post hoc test, WT vs. KO, *p* = 0.005; WT vs. LD, *p* = 0.008; WT vs. HD, *p* = 0.044), but they did not differ between each other (Figure 3h).

### 3.4. Temporal Precision of High-Frequency Signaling Is Supported by Sinbaglustat Treatment

**Figure 4:**
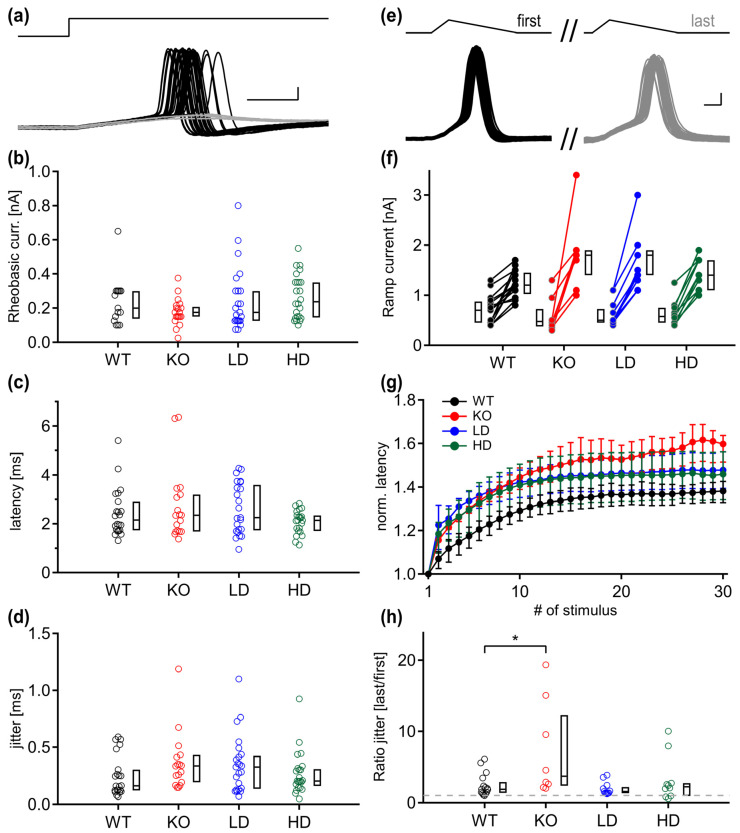
Temporal precision of action potential generation is impaired in *Glb1^−/−^* MNTB neurons during ongoing, high-frequency activity. (**a**) Rheobasic current (top) generating a mix of sub- and suprathreshold responses (gray and black traces, respectively) was injected, and the action potential latency was calculated (bottom). Scale bars: 1 ms, 10 mV. (**b**–**d**) The size of rheobasic current that elicited APs, as well as the AP latency and jitter, did not differ between the animal groups. (**e**) To test the temporal precision during high frequency activity, suprathreshold, 30-pulse stimuli were injected. The superimposed action potentials of the first (black traces) and last stimuli (gray traces) are drawn on the left and right. Scale bars: 0.2 ms, 10 mV. (**f**) Pairwise comparison of ramp current needed to generate a single action potential (left), and current needed to sustain activity (right). (**g**) Median latency values with first and third quartiles during high-frequency stimulation, normalized to the latency of the first spike. Latencies of the KO group do not reach steady state after 30 stimulations, in contrast to the latencies of the WT, LD and HD groups that reach steady state at a similar stimulation step. (**h**) The normalized ratio of jitter, averaged from the last five pulses to the first values, shows that the KO group loses temporal precision. The gray dotted line shows a value of 1. Graphs (**b**–**d**,**h**) show color-coded individual data depending on the experimental group (WT; black, KO; red, low dose (LD); blue, high dose (HD); green) as well as the corresponding boxplots with median, first and third quartile. Statistical significance (Dunn–Bonferroni procedure) was accepted at *p* ≤ 0.05. *; *p* < 0.05.

A hallmark of the mammalian MNTB neurons is their temporal precision of action potential generation. Since the *Glb1^−/−^* phenotype appears to alter the repolarization time, we investigated whether the temporal precision of the input–output functions can be maintained in diseased animals. The square rheobasic current was injected, and the AP latency of suprathreshold events was measured (Figure 4a). Similar to the ramp current (Figure 3b), the median square current was smaller in all *Glb1^−/−^* mice (KO; 175 pA, LD; 175 pA, HD; 237.5) than in WT animals (270 pA) (Kruskal–Wallis, *p* = 0.149) (Figure 4b). No significant difference was found in AP latency (WT; 2.16 ms, KO; 2.36 ms, LD; 2.26 ms, HD; 2.14 ms, Kruskal–Wallis *p* = 0.405) (Figure 4c). A strong trend was observed in the median jitter values, where the KO group had the largest jitter, followed by the LD group, and the jitter of the HD groups was more similar to WT animals (WT; 0.162 ms, KO; 0.336 ms, LD; 0.326 ms, HD; 0.204 ms, Kruskal–Wallis: *p* = 0.054) (Figure 4d). Therefore, the generation of threshold APs appeared largely unaffected.

MNTB neurons are known to sustain high firing frequencies [39,40] with only brief history-dependent adaptation of latency values [37]. To determine whether the temporal precision finally breaks down during sustained activity, we injected a high-frequency train stimulation to generate APs (Figure 4e). The minimum current required for constant firing was calculated and compared with the ramp current that generated single APs (Figure 4f). The current allowing high frequency firing was lowest in the WT group (1.2 nA), followed by the LD and HD groups (1.4 nA), while untreated KO neurons required the highest current (1.8 nA). This difference was only significant between WT and KO mice (Dunn’s post hoc test, WT vs. KO, *p* = 0.046). To visualize latency entrainment, we plotted the normalized AP latency values during the train stimulation of each group. The latency difference during the AP trains reached a plateau in WT, LD and HD groups after about 10 pulses. In the KO group, the latency destabilized and increased throughout the entire AP train (Figure 4g). Comparing the normalized median latency values of the last five action potentials of the animal groups yielded no significant differences. However, we observed that the WT group that had the lowest normalized latency values, along with the LD and HD groups, reached a steady-state adaptation at comparable stimulation steps, contrary to the KO group, where the latencies continued to increase further. Finally, the mean jitter of the last five stimulations of the train was normalized to the first jitter value (Figure 4h). The neurons of the KO group had a higher median value with higher distribution. This ratio significantly differed only between the WT and KO groups (Dunn’s post hoc test, WT vs. KO, *p* = 0.038).

## 4. Discussion

The present study was designed to assess whether sinbaglustat can halt or reverse the impact of G_M1_-gangliosidosis in neurons of the MNTB in a *Glb1^−/−^* knock-out mouse model. Impairment in the auditory system has been reported in different LSDs, such as Fabry disease, Gaucher disease and mucopolysaccharidosis [41]. G_M1_-gangliosidosis disease in humans is categorized into infantile (OMIM# 230500), juvenile (late infantile; OMIM# 230500), and adult (chronic; OMIM# 230650) subtypes. A recent 10-year study on G_M1_-gangliosidosis juvenile/late infantile-type patients revealed that most participants did not show marked changes in peripheral hearing sensitivity. However, using auditory brainstem response testing, juvenile and late infantile patients showed irregularities including decreased wave amplitude, reduced neural synchrony and retrocochlear auditory impairment in the absence of middle ear dysfunction [42]. Therefore, obtained abnormalities seem to emerge from degradation of neural connections in the subcortical acoustic network. Moreover, hyperacusis is a clinical finding in patients suffering from G_M1_ or G_M2_ gangliosidosis [43].

Neurons of the MNTB are affected by hearing impairment. For example, deviations in basal membrane properties were present in a genetic mouse model of early onset hearing loss [44]. In another mouse model for deafness [45], the tonotopic organization of the nucleus was improperly structured. Specifically for the used *Glb1^−/−^* transgenic mice model, MNTB neurons are affected by G_M1_-gangliosidosis and exhibit cytoplasmic enlargement and reduced membrane resistance as compensatory functional mechanisms linked to G_M1_ storage accumulation [16].

The present study showed that sinbaglustat reduced vacuolization when administered at high and low doses, while basal electrophysiological properties of MNTB neurons were unaffected. The WT phenotype of the action potential waveform and temporal precision during high-frequency stimulation of KO neurons were partially maintained after high-dose administration of sinbaglustat.

Purkinje and MNTB neurons of 4-month-old *Glb1^−/−^* mice accumulated lysosomal storage material in vacuoles, which led to somatic enlargement [16]. We confirmed that this accumulation could still be detected in an advanced stage of the disease around 7 months of age with more than half of the cytoplasmic volume of MNTB neurons being occupied by distended lysosomes in untreated *Glb1^−/−^* mice. Long-term administration of sinbaglustat had a positive effect on the reduction of lysosomal storage, but the higher dose did not provide additional benefit. At this later age, we did not detect any significant differences in the somatic capacitance between MNTB neurons of untreated WT and *Glb1^−/−^* mice. One possible explanation for this is that membrane properties change dynamically during disease progression and, at 7 months of age, compensatory mechanisms and increased neuronal variability may cause the capacitance to appear similar to that of WT mice. On the other hand, electroporated and patched MNTB neurons of sinbaglustat-treated *Glb1^−/−^* mice appeared more abundant, had a smoother surface, and survived longer compared with those of non-treated animals. These data corroborate the results of a recent study using histological slices and a semiquantitative score in the same experimental setup [38]. Interestingly, some brain regions, including the cerebellum, showed reduced vacuolization already at low doses of sinbaglustat, whereas comparable results for the diencephalon or midbrain were only observed at high doses [38], with the MNTB investigated in the present study being part of the pons and thus of the midbrain. Furthermore, high-dose application led to a qualitative reduction in Purkinje cell degeneration in the cerebellum, which was interpreted as a possible consequence of prominent GBA2 protein expression in these cells [38]. We concluded that different subtypes of neurons likely react differentially to sinbaglustat, which was reflected in a differentiated therapeutic effect that was more prominent in specific motor areas.

Our electrophysiological results showed that the action potentials (APs) of the *Glb1^−/−^* MNTB neurons were broader, the duration of the after-hyperpolarization was increased, and the temporal precision after fast stimulation was attenuated, compared to WT mice. Animals treated with high-dose application of sinbaglustat had several benefits in these parameters, and their phenotype was not significantly different from the untreated WT group, in contrast to the KO and LD groups. The waveform and kinetics of an AP are crucial for the proper functionality of the circuit, in which MNTB plays an important role. Specifically, brief action potentials are a key factor in maintaining high firing rates and analyzing temporal coding in the MNTB neurons [46]. This temporal precision is crucial for shaping well-timed binaural [47,48] and off-set [49,50,51] responses to sound. The waveform of the action potential is largely shaped by high voltage-activated K^+^ channels that open at voltages reached when an action potential is initiated [52]. In the MNTB, Kv3.1 and Kv3.3 are the main high voltage-activated channels that accelerate action potential repolarization [53,54], and it has been previously demonstrated that blocking these channels with TEA broadens the action potential halfwidth [33,34,55,56]. This pharmacological phenotype resembles the effect observed in the untreated *Glb1^−/−^* mice. Therefore, it might be possible that G_M1_-gangliosidosis also affects the expression of high-voltage activated potassium channels. However, other pathological processes associated with gangliosidosis, such as neuroinflammation, endoplasmic reticulum stress, activation of apoptotic pathways, and disturbances in intracellular Ca^2+^ homeostasis, may also contribute to action potential broadening, either through prolonged depolarization or through alterations in membrane lipid composition.

Modulation of the Kv3.1 and Kv3.3 channels has been associated with deficits in auditory processing and spatial perception, affecting hearing and sound localization [54,57]. Drug treatment on mice with Kv3 channelopathies showed that recovery of physiological Kv3 channel level specifically in the MNTB can improve hearing [58,59]. The importance of fast, stereotyped AP responses of MNTB neurons can be inferred by their inhibitory output to other nuclei of the superior olivary complex, namely the lateral superior olive (LSO) and the superior paraolivary nucleus (SPN). The LSO processes interaural level differences (ILDs) by comparing the contralateral inhibition via the MNTB and the excitation from the ipsilateral cochlear nucleus [47,60]. Longer lasting inhibition from the MNTB, as is the case in untreated *Glb1^−/−^* mice, could shift the sigmoid ILD sensitivity and compromise sound localization. The SPN is implicated in gap detection [50,51] and the SPN neurons fire an offset response only after the strong inhibition of the MNTB is lifted. Therefore, longer glycinergic APs may integrate the sound duration incorrectly and affect the temporal computation of higher nuclei.

Overall, this electrophysiological study presented certain difficulties and limitations. The MNTB neurons of the *Glb1^−/−^* mice were subjectively of a lower quality than the other groups. Many were dead, and most of them had a very rough surface and could not be efficiently patched. Additionally, the sinbaglustat-treated groups had a phenotype closer to the WT in various parameters, although they did not reach significance due to high variability. Finally, we noticed that *Glb1^−/−^* MNTB neurons still retained a higher degree of functionality than we expected, demonstrating the robustness of this network. Investigating another system, such as the cerebellum, might have provided more evident differences between the animal groups.

In summary, we detected subtle electrophysiological abnormalities in MNTB neurons of *Glb1^−/−^* mice, regarding the AP waveform and temporal precision. Sinbaglustat reduces vacuolization in these neurons, but its effect is saturated already in low-dose-treated animals. This reduction might facilitate the maintenance of the correct phenotype.

## 5. Conclusions

The present study demonstrates that neurons of the MNTB in *Glb1*^−/−^ mice exhibit structural and electrophysiological alterations associated with storage material accumulation. Long-term treatment with sinbaglustat reduced lysosomal storage and partially preserved the action potential waveform and temporal fidelity, especially at a higher dose. These findings support the potential of GBA2 inhibition to alleviate neuronal dysfunction in G_M1_-gangliosidosis, while also indicating that therapeutic responses may vary across neuronal populations and stages of the disease.

## Data Availability

The datasets generated and analyzed during the current study are available from the corresponding author on reasonable request.

## Abbreviations

The following abbreviations are used in this manuscript:

GBA2	Non-lysosomal β-galactosidase 2
GCS	Glutamate–cysteine ligase
MNTB	Medial Nucleus of the Trapezoid Body
LD	Low-Dose
HD	High-Dose
KO	Knock-Out
WT	Wild Type
LSD	Lysosomal Storage Disease
β-gal	β-galactosidase
SRT	Substrate Reduction Therapy
AP	Action Potential
LAVES	Niedersächsisches Landesamt für Verbraucherschutz und Lebensmittelsicherheit
LJP	Liquid junction potential
C	Capacitance
ROI	Region of Interest
E_rest_	Resting Potential
R_in_	Input Resistance
τ_mem_	Membrane time constant
V_thres_	Voltage threshold
